# Evaluating longitudinal cytomegalovirus-specific humoral immune responses and association with DNAemia risk in seropositive lung transplant recipients

**DOI:** 10.1016/j.jhlto.2024.100113

**Published:** 2024-05-28

**Authors:** Melissa J. Harnois, Richard Barfield, Maria Dennis, Nicole Rodgers, Justin Pollara, Connor S. Spies, Laurie D. Snyder, Cliburn Chan, Annette M. Jackson, Scott M. Palmer, Sallie R. Permar

**Affiliations:** a Department of Immunology, Duke University School of Medicine, Durham, North Carolina; b Duke Human Vaccine Institute, Duke University Medical Center, Durham, North Carolina; c Department of Surgery, Duke University School of Medicine, Durham, North Carolina; d Department of Biostatistics and Bioinformatics, Duke University School of Medicine, Durham, North Carolina; e Center for Human Systems Immunology, Duke University Medical Center, Durham, North Carolina; f Department of Pediatrics, Weill Cornell Medicine, New York, New York; g Division of Pulmonary, Allergy and Critical Care, Duke University School of Medicine, Durham, North Carolina

**Keywords:** cytomegalovirus, CMV, lung transplant, humoral immunity, CMV DNAemia

## Abstract

**Background:**

Cytomegalovirus (CMV) is the most common viral infection among lung transplant recipients and is associated with chronic lung allograft dysfunction. There is a need for better therapeutics as well as biomarkers to enable effective stratification of CMV seropositive patient risk for developing CMV DNAemia to inform prophylaxis duration.

**Methods:**

CMV-specific immunoglobulin G (IgG) binding and functional responses were evaluated in a discovery cohort of longitudinal plasma samples from 51 CMV seropositive human lung transplant recipients, collected as part of the clinical trials in organ transplantation (CTOT)-20 and CTOT-22 consortium studies. Pre-transplant plasma from an additional 43 CMV seropositive lung transplant recipients was evaluated as a validation cohort.

**Results:**

In the discovery cohort with longitudinal samples, pre-transplant plasma IgG binding to CMV surface glycoproteins glycoprotein H (gH)/glycoprotein L (gL), gH/gL/glycoprotein O (gO), and pentameric complex, as well as neutralization of CMV in epithelial cells, is associated with increased risk of CMV DNAemia post-prophylaxis. However, these results were not confirmed by the validation cohort.

**Conclusions:**

While quantification of pre-transplant CMV-specific antibody responses showed association with DNAemia in the discovery cohort, additional clinical variables and/or known risk factors for CMV, such as patient CMV-specific T-cell responses, may need to be considered in combination with humoral immunity to effectively stratify risk of CMV DNAemia.

## Background

Cytomegalovirus (CMV) is a ubiquitous herpesvirus that establishes life-long infection in humans and is the most common viral cause of post-transplant disease.^1^ Lung transplant (LT) patients are at particularly high risk for developing CMV-associated disease due to prolonged T-cell immunosuppression and the relatively large volume of transplanted lymphoid tissue.^2^, ^3^, ^4^ Importantly, CMV DNAemia is an independent risk factor for chronic lung allograft dysfunction, the leading complication limiting long-term survival in lung transplantation.^5^, ^6^, ^7^ Thus, preventing the development of CMV DNAemia in LT recipients is critical to improving allograft and patient survival.

Risk for CMV DNAemia is assessed based on the serostatus of the organ donor (D) and recipient (R). CMV seronegative recipients of an organ from a CMV seropositive donor (R-/D+) are at highest risk for developing CMV-associated disease, while CMV seronegative recipients of an organ from a CMV seronegative donor (R-/D-) are at lowest risk. CMV seropositive recipients (R+) are considered “intermediate risk” due to their pre-existing CMV-specific immunity. Prophylactic antiviral drugs are highly effective at suppressing viral replication, but their long-term use is not ideal due to toxicities.^8^ Regardless of prophylaxis duration, approximately 45% of R+ LT patients develop CMV DNAemia upon cessation of antivirals, which is the highest incidence across solid organ transplant types.^8^, ^9^, ^10^ The factors that determine which R+ patients will develop CMV DNAemia post-prophylaxis remain unclear, highlighting a critical need for novel biomarkers to enable effective risk stratification of R+ patients and a more individualized approach to antiviral treatment.

Recent studies that have highlighted the potential value of monitoring patient immune status to predict the risk of CMV DNAemia and disease have focused primarily on CMV-specific cellular immunity.^11^, ^12^, ^13^ Many assays have been developed to assess CMV-specific T-cell responses, though these methods remain generally limited in their ability to stratify patient risk of CMV DNAemia or disease and are not routinely used in clinical practice.^14^, ^15^ Few studies have focused on the role of CMV-specific humoral immunity in LT. Clinical serology tests are used to determine whether a patient has been infected with CMV, and quantitative polymerase chain reaction is used to detect and monitor replicating virus in the blood. These tests evaluate a single timepoint and do not provide insight into the immune status of seropositive patients over time. Thus, in this study, we evaluated CMV-specific antibody responses in plasma collected pre-transplantation through 12 months post-transplantation from CMV seropositive LT recipients. We measured antibody binding to whole CMV virions and viral glycoproteins that are known targets of the anti-CMV humoral response. Additionally, we evaluated CMV-specific antibody functional responses, including neutralization, antibody-dependent cellular phagocytosis (ADCP), and antibody-dependent cellular cytotoxicity (ADCC). This project aims to advance our understanding of CMV-specific humoral immunity in lung transplantation to improve risk stratification and to better understand the role of anti-CMV humoral immunity in transplant-related CMV disease outcomes.

## Materials and methods

### Study approvals

This project utilized longitudinally collected plasma from CMV seropositive adult human LT recipients from the clinical trials in organ transplantation (CTOT)-20 and CTOT-22 studies^16^ as well as from Duke University. The use of samples and relevant clinical data for this project was approved by the Duke University Institutional Review Board (Pro00106673, Pro00109661, Pro00104220).

### Discovery cohort

The discovery cohort was comprised of *n* = 44 CMV seropositive patients from the CTOT-22 study and an additional 7 CMV seropositive CTOT-20 patients from Duke who developed CMV DNAemia. The additional 7 patients were selected using CTOT-22 inclusion criteria.^16^ Patient plasma samples were collected at 0, 2, 3, 6, 9, and 12 months post-transplantation. Samples were not available for all patients at every timepoint; 56% of patients (29 of 51) had available pretransplant samples and 88% to 100% of patient samples were available at each subsequent timepoint. Within the discovery cohort, 47% (24 of 51) developed CMV DNAemia within 15 months of removing antiviral prophylaxis. The duration of antiviral prophylaxis and monitoring for CMV DNAemia were based on the center’s practice. Prophylaxis was either 6 or 12 months post-transplantation per center protocol and monitoring was performed at regular intervals per center protocol. Demographics of the validation cohort are detailed in Table 1.

**Table 1:** Discovery Cohort Demographic Data

Variable	CMV DNAemia (N = 24)	No CMV DNAemia (N = 27)
Age		
Mean (SD)	55.8 (13.8)	58.1 (11.7)
Median [min, max]	61.5 [21.0, 73.0]	60.0 [22.0, 69.0]
Sex		
Female	13 (54.2%)	11 (40.7%)
Male	11 (45.8%)	16 (59.3%)
Race		
American Indian or Alaska Native	1 (4.2%)	1 (3.7%)
Asian	3 (12.5%)	0 (0%)
Black or African American	3 (12.5%)	2 (7.4%)
White	17 (70.8%)	23 (85.2%)
Unknown or not reported	0 (0%)	1 (3.7%)
Type of transplant		
Bilateral	22 (91.7%)	20 (74.1%)
Single	2 (8.3%)	7 (25.9%)
Donor CMV serostatus		
D−/R+	4 (16.7%)	14 (51.9%)
D+/R+	20 (83.3%)	13 (48.1%)
Days off prophylaxis		
Mean (SD)	300 (145)	314 (110)
Median [min, max]	284 [122, 679]	362 [107, 508]
Event		
CMV	24 (100%)	0 (0%)
End of study	0 (0%)	8 (29.6%)
End of study, extension study	0 (0%)	18 (66.7%)
Transfer	0 (0%)	1 (3.7%)

Abbreviation: CMV, cytomegalovirus; D+, CMV seropositive organ donor; D−, CMV seronegative organ donor; R+, CMV seropositive organ recipient; R− CMV seronegative organ recipient; SD, standard deviation.

### Validation cohort

The validation cohort was comprised of only pre-transplant plasma samples from Duke University patients selected using CTOT-22 inclusion criteria during the CTOT-22 enrollment period.^16^ The validation cohort focuses on pre-transplant samples because this was the only timepoint at which an association between CMV-specific antibody responses and DNAemia post-prophylaxis was identified in the discovery cohort. Demographics of the validation cohort are detailed in Table 2.

**Table 2:** Validation Cohort Demographic Data

Variable	CMV DNAemia/death (N = 23)	No CMV DNAemia (N = 20)
Age		
Mean (SD)	63.5 (8.06)	58.2 (10.0)
Median [min, max]	63.0 [43.0, 75.0]	59.5 [29.0, 73.0]
Sex		
Female	10 (43.5%)	6 (30.0%)
Male	13 (56.5%)	14 (70.0%)
Race		
Black or African American	5 (21.7%)	0 (0%)
White	18 (78.3%)	20 (100%)
Type of transplant		
Bilateral	20 (87.0%)	18 (90.0%)
Single	3 (13.0%)	2 (10.0%)
CMV serology		
D−/R+	6 (26.1%)	8 (40.0%)
D+/R+	17 (73.9%)	12 (60.0%)
Days off prophylaxis		
Mean (SD)	379 (79.5)	430 (88.1)
Median [min, max]	371 [216, 573]	413 [267, 608]
Study		
CTOT-20	10 (43.5%)	20 (100%)
Duke patients	13 (56.5%)	0 (0%)
Event		
CMV	21 (91.3%)	0 (0%)
Death	2 (8.7%)	0 (0%)
End of study	0 (0%)	19 (95.0%)
Transfer	0 (0%)	1 (5.0%)

Abbreviation: CMV, cytomegalovirus; CTOT, clinical trials in organ transplantation; D+, CMV seropositive organ donor; D−, CMV seronegative organ donor; R+, CMV seropositive organ recipient; R− CMV seronegative organ recipient; SD, standard deviation.

#### Whole virion enzyme-linked immunosorbent assay

Plasma antibody binding and avidity for CMV strain AD169r and linear glycoprotein B (gB)-AD2 peptide were measured by enzyme-linked immunosorbent assay (ELISA) as previously described.^17^ Briefly, plates were coated with virus (2,700 plaque forming units/well) or peptide (2 µg/ml) and incubated at 4°C overnight. Plates were washed, blocked, and incubated overnight. Plates were washed and samples were added in duplicate for 2 hours at room temperature (RT). For the immunoglobulin G (IgG) avidity measurement only, plates were washed and 7M urea was added to half of the wells for 8 minutes at RT. Plates were washed and secondary antibody (goat antihuman IgG-HRP or goat antihuman IgM-HRP, Jackson ImmunoResearch or biotinylated IgG subclass-specific secondary antibodies, Southern BIotech) was added for 1 hour. For the IgG1, IgG2, IgG3, and IgG4 subclass assays only, plates were washed, and horseradish peroxidase (HRP)-conjugated streptavidin (Jackson ImmunoResearch) was added for 15 minutes. Plates were washed and SureBlue Reserve TMB Substrate (VWR) was added for 10 minutes, followed by TMB Stop Solution (VWR). Plates were read at 450 nm on the SpectraMax plate reader (Molecular Devices). Results are reported as area under the curve because full sigmoidal curves were not achieved by all samples. For the IgG subclass assay, serial dilutions of IgG subclass proteins purified from human myeloma plasma (Sigma) were used to create standard curves. Cytogam (CSL Behring) or previously characterized immunoglobulin M (IgM)-positive human plasma samples were used as positive controls to ensure consistent performance across runs. CMV seronegative plasma samples were included as negative controls. Negative cutoffs were established based on the average seronegative area under the curve plus 3× standard deviation.

#### Binding antibody multiplex assay

Antibody binding to purified, soluble CMV surface glycoproteins (gB Ectodomain, pentameric complex (PC), gH/gL, and gH/gL/gO) were measured via multiplex assay as previously described.^17^ Proteins were covalently coupled to fluorescent polystyrene beads (Luminex) and incubated with plasma samples for 30 minutes. Samples were washed and incubated with phycoerythrin-conjugated goat antihuman IgG for 30 minutes (2 µg/ml, Southern Biotech). Beads were washed and mean fluorescence intensity was measured on a Bio-Plex 200 Plate Reader (Bio-Rad). Cytogam (CSL Behring) and CMV seronegative plasma samples were included as positive and negative controls, respectively. All samples and controls were tested in duplicate. Negative cutoffs were established for each antigen based on the average mean fluorescence intensity of seronegative samples plus 3× the standard deviation.

#### CMV neutralization

Human foreskin fibroblast (HFF1) cells or human acute retinal pigment epithelial (ARPE) were plated at 2,000 (ARPE) or 3,000 (HFF1) cells per well and incubated for 24 (ARPE) or 48 (HFF1) hours at 37°C. Serum samples and controls were serially diluted and incubated with CMV AD169r-GFP virus (HFF1, multiplicity of infection (MOI) of 1.5) or AD169r untagged virus (ARPE, MOI of 2) at 37°C for 1 hour. Samples and viruses were added to cells for 20 (HFF1) or 48 hours (ARPE) at 37°C. Cells were fixed and stained with mouse anti-CMV immediate-early 1 antibody (Abcam) for 1 hour at 37°C. Cells were washed and goat antihuman AF488 (Abcam) was added for 55 minutes at 37°C, followed by 4'6-diamidino-2-phenylindole (Thermo Fisher) for 10 minutes at RT. Wells were washed and resuspended in phosphate buffered saline (PBS). Plates were sealed and imaged on the CellInsight CX5 High Content Screening Platform (Thermo Fisher). The sample dilution corresponding to neutralization of half of the maximum infection (NT50) was calculated in GraphPad Prism v9.0.

#### Antibody-dependent cellular phagocytosis

AD169r virus was conjugated to AF647 NHS ester (Invitrogen) and incubated for 2 hours with diluted plasma and controls at 37°C. Next, 50,000 THP-1 cells were added to each well, and plates were centrifuged at 1,200×g at 4°C for 1 hour, incubated at 37°C for 1 hour, then centrifuged again at 1,200×g for 5 minutes. Media was removed and Aqua live/dead stain was added for 15 minutes. Cells were washed, fixed, and resuspended in PBS for acquisition on the BD Fortessa using the high throughput sampler input. Cytogam was used as a positive plate control, known seronegatives, a nonspecific monoclonal antibody, and conjugated virus-only wells were included as negative controls for the assay.

#### Natural killer cell CD107a degranulation ADCC assay

Cell-surface expression of CD107a was measured as a marker for natural killer (NK) cell degranulation^18^, ^19^ as previously described.^17^ Live primary human NK cells were added to wells containing AD169-derivative BadrUL131-Y4-GFP–infected MRC-5 cell monolayers. Diluted plasma samples were added with brefeldin A (GolgiPlug), monensin (GolgiStop), and anti–CD107a-fluorescein isothiocyanate for 6 hours. Next, NK cells were washed and stained with anti–CD56-phycoerythrin/cyanine 7 viability dye and anti–CD16-PacBlue.^20^ The frequencies of CD107a+ live NK cells were measured by flow cytometry. Final data represent specific activity, calculated by subtraction of nonspecific activity observed in assays performed with mock-infected cells.

#### Statistical analysis

The statistical analysis plan was created a priori. Raw data were reviewed and verified before analysis using established quality control metrics for each assay. Next, we analyzed each variable’s association with CMV DNAemia-free survival with Cox-proportional hazards model separately at pretransplant, 2 to 3 months post-transplant while on prophylaxis, and the post-transplant observation closest to prophylaxis stoppage. In the latter 2 analyses, we adjusted for age. Multiple testing correction was done separately within each of the analyses, with statistical significance predefined as *p* < 0.05, with a false discovery rate (FDR)-adjusted *p*-value of <0.2, reflecting the exploratory nature of this study.^21^ We scaled each variable to have a mean 0 and variance 1 before analysis. All statistical analyses were performed in R.22., 23

## Results

### Patients who develop CMV DNAemia appear to exhibit differing patterns of longitudinal plasma antibody binding to whole CMV virions, relative to patients who do not develop DNAemia

**Figure 1:**
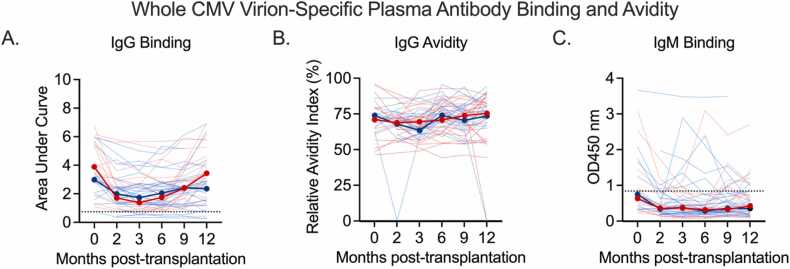
CMV-specific plasma antibody binding to AD169r. Plasma IgG (A) IgG binding, (B) IgG avidity, and (C) IgM binding to whole CMV AD169r virions pre-transplantation (0 months) through 12 months post-transplantation in LT recipients who did not develop CMV DNAemia (blue) and who did develop CMV DNAemia (red) within 15 months of stopping antiviral prophylaxis. Thin lines show individual patient data and thick red and blue lines indicate median response. Dotted lines indicate the negative cutoff, established by the mean plus 3× standard deviation of CMV seronegative plasma samples. CMV, cytomegalovirus; IgG, immunoglobulin G; IgM, immunoglobulin M; LT, lung transplant.

We first describe the longitudinal pattern of plasma antibodies. This is purely descriptive with no statistical analyses as these patterns occur before the event of interest. CMV-specific plasma IgG and IgM binding responses were measured longitudinally by whole virion ELISA (Figure 1). We observed that patients who did not develop CMV DNAemia (no CMV infection, CMVi−)^24^ maintained relatively stable levels of CMV-specific plasma IgG throughout the study period, while patients who did develop CMV DNAemia (CMV infection, CMVi+)^24^ exhibited elevated CMV-specific plasma IgG binding pre-transplantation, followed by lower binding responses between 2 and 3 months post-LT, and finally rebound to near pre-transplant levels between 6 and 12 months post-LT (Figure 1A). Both groups exhibited similar and consistent levels of plasma IgG avidity for the whole CMV virion over time (Figure 1B), and 40% of patients (20 of 51) had detectable levels of CMV-specific plasma IgM at least once during the study (Figure 1C). Of the 20 patients with CMV-specific IgM, approximately half came from each group (9 CMVi− vs 11 CMVi+).

Whole virion ELISA was also used to quantify CMV-specific IgG subclass concentration in patient plasma (Figure S1). As expected, the majority of detectable CMV-specific IgG in plasma were of IgG1 and IgG3 subclass. Twelve patients had detectable CMV-specific IgG2 and no patients had detectable IgG4. IgG1 concentration decreased in both groups between 0 and 2 months post-transplantation (CMVi− group: 8.5% median decrease in 11 patients with samples at both timepoints; CMVi+ group: 13.8% decrease in 14 participants with samples at both timepoints, all shifted by 1 to account for 0). The CMVi− median post-transplant IgG1 concentration remained relatively stable for the remainder of the study period, while the median IgG1 concentration of the CMVi+ group increased by 9.6% between the 9- and 12-month timepoints in the 23 patients with data at both timepoints. The median CMV-specific IgG3 concentrations remained much lower than that of IgG1, as expected, and both groups exhibited similar median CMV-specific plasma IgG3 concentrations throughout the study period.

### Patients with CMV DNAemia appear to exhibit differing patterns of longitudinal plasma antibody binding to CMV surface glycoproteins, relative to patients without CMV DNAemia

**Figure 2:**
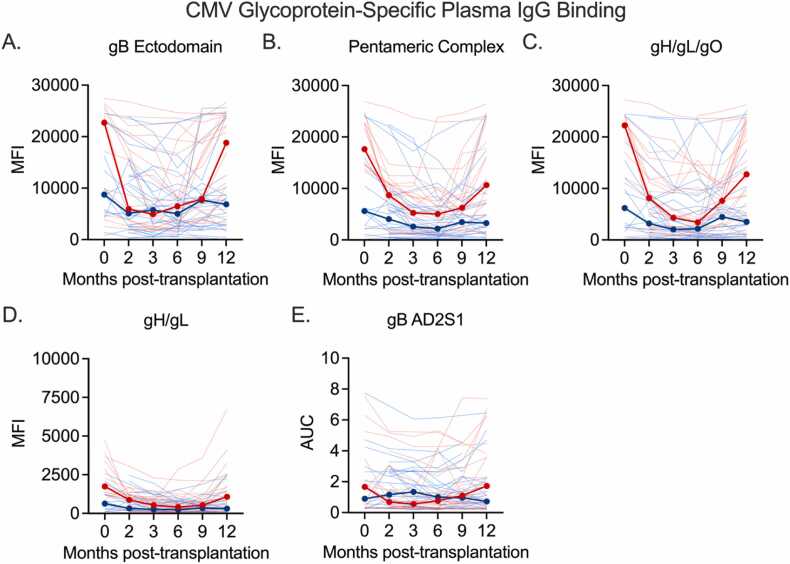
Plasma IgG binding to CMV surface glycoproteins. (A) Glycoprotein B (gB), (B) pentameric complex (PC), (C) gH/gL dimer, (D) gH/gL/gO trimer, (E) gB-AD2S1 pre-transplantation in lung transplant recipients who did not develop CMV DNAemia (blue) and who did develop CMV DNAemia (red) within 15 months of stopping antiviral prophylaxis. Thin lines show individual patient data and thick red and blue lines indicate median response. AUC, area under the curve; CMV, cytomegalovirus; MFI, mean fluorescence intensity.

Next, we measured CMV surface glycoprotein-specific IgG binding levels. Similar to the trend observed in the whole virion ELISA, CMVi+ patients exhibit high median IgG binding responses to gB, PC, gH/gL, and gH/gL/gO pre-transplantation, followed by low-level IgG binding from 2 to 6 months post-LT, and a subsequent increase in binding between 6 and 12 months post-transplantation (Figure 2A-D). CMVi− patients also exhibit a modest decrease in median glycoprotein-specific IgG binding from pre- to post-transplant, but do not experience the IgG rebound at later timepoints which is observed among CMVi+ patients (Figure 2A-D). Interestingly, while the median CMVi+ plasma IgG binding to neutralizing epitope gB-AD2 site 1 (gB-AD2S1) follows the same trend observed for other glycoproteins, CMVi− patient plasma exhibits an increase in IgG binding to AD2S1 between 0 and 3 months post-transplantation, though not statistically significant (Figure 2E).

### Plasma CMV-specific antibody functional responses are comparable among patients who did and did not develop CMV DNAemia

**Figure 3:**
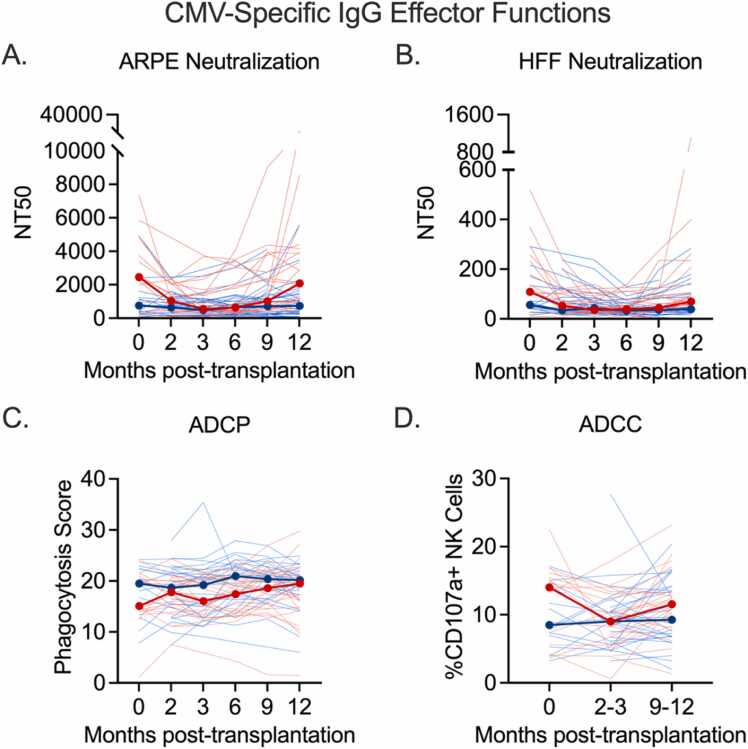
CMV-specific plasma antibody effector functions. CMV neutralization on (A) acute retinal pigment epithelial (ARPE) and (B) human foreskin fibroblast (HFF) cells and non-neutralizing effector functions (C) antibody-dependent cellular phagocytosis and (D) antibody-dependent cellular cytotoxicity, were measured in plasma from LT recipients who did not develop CMV DNAemia (blue) and who did develop CMV DNAemia (red) within 15 months of stopping antiviral prophylaxis. Thin lines show individual patient data and thick red and blue lines indicate median response. CMV, cytomegalovirus; LT, lung transplant; ADCC, antibody-dependent cellular cytotoxicity; ADCP, antibody-dependent cellular phagocytosis; NK, natural killer.

In addition to binding responses, CMV neutralization on epithelial cells and fibroblasts, ADCP, and ADCC were evaluated. ADCC responses and CMV neutralization on fibroblasts and epithelial cells mirrored the response trends observed in both groups for IgG binding to the whole virion and surface glycoproteins. Interestingly, median ADCP responses were consistently higher in the CMVi− group, relative to the CMVi+ group, throughout the study (Figure 3).

### Assessment of patient risk for developing CMV DNAemia post-prophylaxis reveals that elevated pre-transplant antibody binding and functional responses are associated with increased risk of CMV DNAemia post-prophylaxis

**Figure 4:**
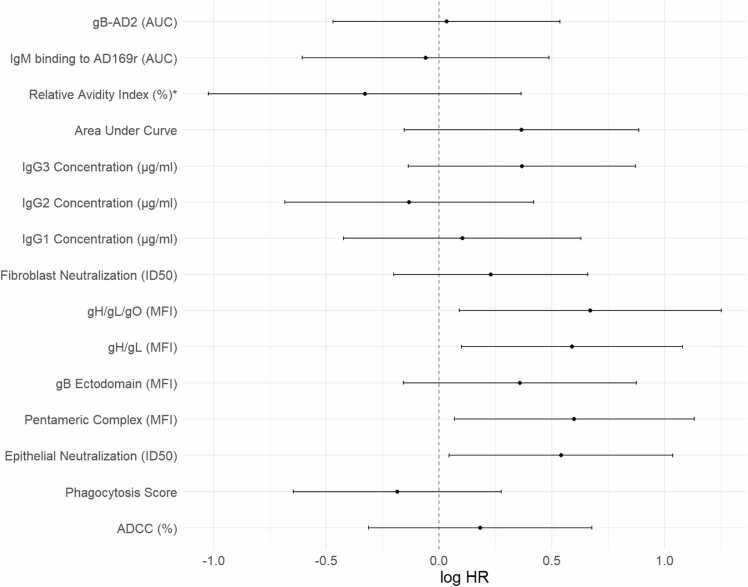
Cox-proportional hazards risk for developing CMV DNAemia post-transplantation. Analysis of pretransplant antibody binding and functional responses by Cox-proportional hazard to assess the risk of developing CMV DNAemia post-transplantation. Statistical significance was defined as *p* < 0.05 with false discovery rate (FDR) <0.2. ADCC, antibody-dependent cellular cytotoxicity; AUC, area under curve; CMV, cytomegalovirus; gB, glycoprotein B; MFI, mean fluorescence intensity.

Patient risk for developing CMV DNAemia post-transplantation would ideally be assessed before transplantation for early risk stratification of CMV seropositive patients. Thus, we ran a Cox-proportional hazards analysis utilizing testing the association between each measured variable and CMV DNAemia-free survival (Figure 4, Table S1). We found that patients with elevated pretransplant antibody binding to CMV PC (*p* = 0.0271, FDR*p* = 0.122), gH/gL (*p* = 0.0184, FDR*p* = 0.122), and gH/gL/gO (*p* = 0.0237, FDR*p* = 0.122), and neutralization of CMV on epithelial cells (*p* = 0.0325, FDR*p* = 0.122) exhibited the shortest time to DNAemia or death after stopping prophylaxis. This suggests that these elevated pretransplant binding and neutralization responses are associated with a higher risk of developing CMV DNAemia postprophylaxis. No other variables were adjusted for in this analysis due to the small sample size. Two posthoc sensitivity analyses were performed on the statistically significant variables, one adjusting for age and another separately for donor serostatus. Upon adjusting for age, all variables remained statistically significant (Table 3). None of the variables remained statistically significant upon adjusting for donor CMV serostatus, likely due to the small sample size (Table 4). However, the direction and magnitude of the effect estimates remained similar (Figure S2). There is some evidence in the literature suggesting D+ status can influence R+ outcomes,^3^, ^25^, ^26^ though the impact of pre-existing immunity in these distinct groups may require larger cohorts to discern in LT. None of the analyses at later timepoints were found to be significant.

**Table 3:** Sensitivity Analysis Adjusting for Recipient Age

Variable	Log HR estimate	SE	*p*-value
ARPE neutralization	0.765	0.318	0.0160
gH/gL/gO	0.654	0.303	0.0308
gH/gL	0.556	0.260	0.0323
PC	0.583	0.278	0.0359

Abbreviations: ARPE, acute retinal pigment epithelial; HR, hazards ratio; PC, pentameric complex.

**Table 4:** Sensitivity Analysis Adjusting for Donor Serostatus

Variable	Log HR estimate	SE	*p*-value
gH/gL	0.471	0.278	0.0901
gH/gL/gO	0.455	0.318	0.1520
PC	0.368	0.293	0.2090
ARPE neutralization	0.318	0.260	0.2220

Abbreviations: ARPE, acute retinal pigment epithelial; PC, pentameric complex.

In a separate exploratory analysis, we used a Cox model to investigate if the relative change in response from pre-transplantation to early post-transplantation is associated with DNAemia risk. No variables were found to be significant, as shown in Table S2.

### Analysis of potential biomarkers of CMV DNAemia in a validation cohort of CMV seropositive LT recipients

**Figure 5:**
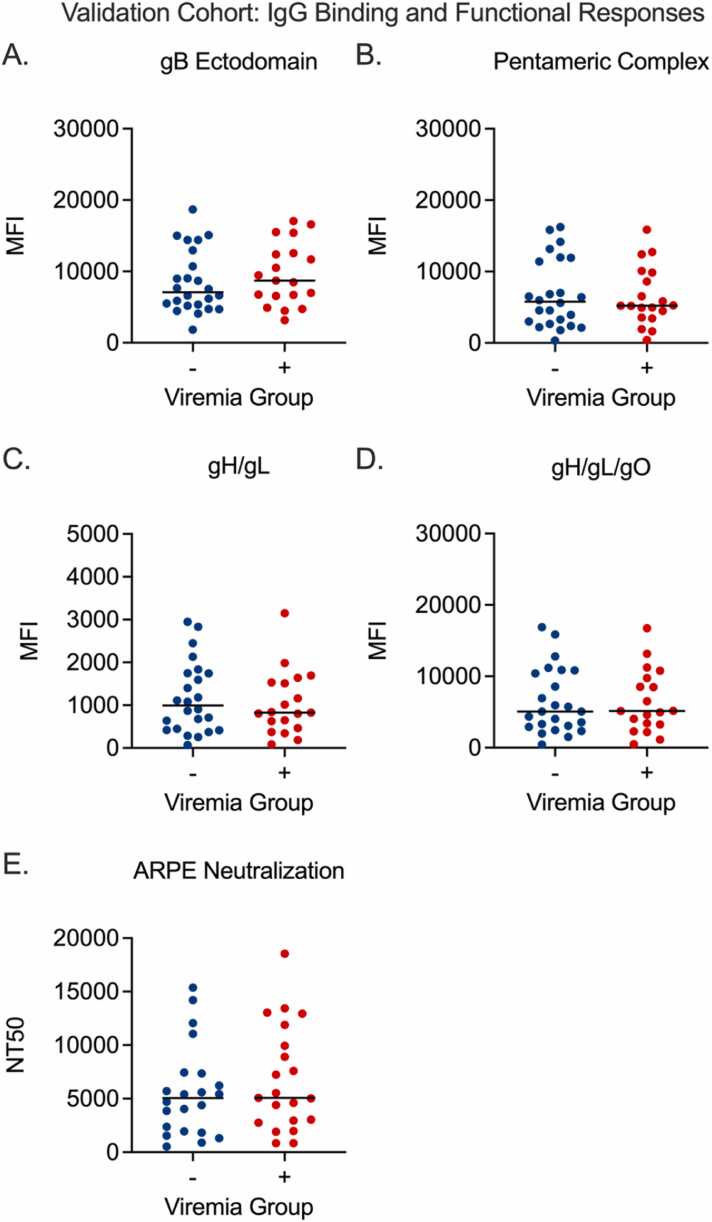
Validation cohort IgG binding and functional responses. (A) Glycoprotein B (gB), (B) pentameric complex (PC), (C) gH/gL dimer, (D) gH/gL/gO trimer, and (E) neutralization of CMV on acute retinal pigment epithelial (ARPE) cells were measured in a validation cohort of *n* = 40 LT recipients who did not develop CMV DNAemia (blue, *n* = 20) and who did develop CMV DNAemia (red, *n* = 20) within 15 months of stopping antiviral prophylaxis. Black line indicates the median response. CMV, cytomegalovirus; LT, lung transplant; MFI, mean fluorescence intensity.

A validation cohort (*n* = 43) was selected from CMV seropositive LT recipients within Duke University that matched the CTOT-22 enrollment criteria and enrollment period to minimize differences between the discovery and validation cohorts. Pretransplant plasma samples were obtained and evaluated for CMV-glycoprotein–specific IgG binding and CMV neutralization on epithelial cells (Figure 5). All data were analyzed using the same Cox-proportional hazards model to determine the association of each measured variable with DNAemia-free survival upon cessation of antiviral prophylaxis. None of the variables found to be associated with increased risk of CMV DNAemia at the pretransplant timepoint in the discovery cohort replicated in the validation cohort (Figure 5, Table S3).

## Discussion

The present study characterized longitudinal anti-CMV antibody specificity and functional responses in CMV seropositive LT recipient plasma. We observed different patterns of CMV-specific IgG dynamics over time in plasma of LT patients who developed CMV DNAemia after prophylaxis ended, in comparison to patients who did not develop DNAemia. The same pattern has been reported in other recent longitudinal analyses of solid organ transplant recipient plasma antibodies with specificity for CMV and severe acute respiratory syndrome coronavirus 2 (SARS-CoV-2).^27^, ^28^ Surprisingly, results from the discovery cohort revealed that elevated pre-transplant IgG binding responses against CMV surface glycoproteins gH/gL, gH/gL/gO, and the PC, as well as neutralization of CMV on epithelial cells, are associated with an increased risk of developing CMV DNAemia post-prophylaxis. However, the validation cohort did not replicate this finding. These conflicting results between the discovery and validation cohorts highlight a major challenge of identifying biomarkers within human populations and the complexity of patient-specific factors that may contribute to DNAemia risk in lung transplantation.

The PC is required for entry into epithelial and endothelial cells and is targeted by the most potently neutralizing CMV-specific antibodies. Although most antibodies elicited by natural CMV infection are gB-specific, elevated levels of pre-transplant antibodies against gB were not significantly associated with increased risk of DNAemia in this study. Further, a gB subunit vaccine achieved partial efficacy in reducing CMV DNAemia in renal transplant recipients, possibly by enhancing non-neutralizing anti-gB IgG responses.^29^ While we did not observe a statistically significant association between DNAemia risk and non-neutralizing responses, longitudinal ADCP responses were consistently higher among patients who did not develop CMV DNAemia, relative to the CMVi+ group. Recent studies have associated CMV-specific ADCP responses with vaccine-mediated protection from CMV acquisition,^20^ reduced risk of congenital CMV infection,^30^ as well as with protection from DNAemia in seronegative LT recipients.^31^

This study has several limitations. First, this cohort of LT recipients was relatively small in size to identify biomarkers in a population with heterogeneous clinical risk factors. The analysis did not account for single vs bilateral LT, which has the potential to impact patient risk for developing CMV DNAemia in cases where D+ introduces new strains of CMV to the recipient and/or greater antigenic load. However, most patients included in this study received a bilateral LT and these were equally distributed across DNAemia groups in the discovery and validation cohorts. Further, we did not account for any deviations in immunosuppression regimen, a key factor in CMV DNAemia risk after transplantation, and the validation cohort was comprised of only patients from Duke. Although the CTOT-22 inclusion criteria were followed, some centers stop prophylaxis after 6 months whereas Duke patients remain on prophylaxis for 12 months.

Identification of immune correlates of protection from CMV remains challenging in all clinical contexts due to the coevolution of CMV and the human immune system over millennia. This study demonstrates that CMV antibody binding and functional responses have the potential to be used as biomarkers for CMV DNAemia risk, but not interpreted as immune responses that may be more adept at containing DNAemia after lung transplantation. Monitoring the immune status of R+ patients by measuring CMV-glycoprotein–specific IgG levels, in combination with other known risk factors for DNAemia, such as T-cell responses, is one potential strategy for advancing individualized risk stratification and decision-making regarding antiviral prophylaxis treatment duration.^14^, ^32^, ^33^ Additional studies in larger cohorts of CMV seropositive LT recipients will be required to establish a multifactorial approach to risk stratification to effectively mitigate CMV DNAemia via individualized antiviral treatment regimens. Decreasing the risk of CMV DNAemia will improve allograft and patient outcomes by decreasing the risk of chronic lung allograft dysfunction, morbidity, and mortality.

## Disclosure statement

The authors declare the following financial interests/personal relationships which may be considered as potential competing interests: Melissa J. Harnois reports financial support was provided by the 10.13039/100000060National Institute of Allergy and Infectious Diseases T32 (2T32AI052077-16A1). Cliburn Chan reports financial support was provided by 10.13039/100000002NIH U01AI113315. Scott M. Palmer reports financial support was provided by the National Institute of Allergy and Infectious Diseases (U01AI113315). Scott M. Palmer reports financial support was provided by 10.13039/100000897Cystic Fibrosis Foundation (PALMER19AB0). Scott M. Palmer reports a relationship with Incyte that includes funding grants. Scott M. Palmer reports a relationship with 10.13039/100004325AstraZeneca that includes funding grants. Scott M. Palmer reports a relationship with 10.13039/100008021Bristol Myers Squibb that includes funding grants and speaking and lecture fees. Scott M. Palmer reports a relationship with 10.13039/100016477CareDx that includes funding grants. Scott M. Palmer reports a relationship with Boehringer Ingelheim Pharmaceuticals Inc. that includes funding grants and speaking and lecture fees. Scott M. Palmer reports a relationship with Altavant Sciences, Inc. that includes speaking and lecture fees. Scott M. Palmer reports a relationship with Mallinckrodt Pharmaceuticals that includes speaking and lecture fees. Scott M. Palmer reports a relationship with Natera that includes speaking and lecture fees. Sallie Permar reports a relationship with Merck Vaccines that includes consulting or advisory and funding grants. Sallie Permar reports a relationship with Moderna Inc. that includes consulting or advisory and funding grants. Sallie Permar reports a relationship with Dynavax that includes consulting or advisory. Sallie Permar reports a relationship with Pfizer Inc. that includes consulting or advisory. Sallie Permar reports a relationship with GlaxoSmithKline Inc. that includes consulting or advisory. Sallie Permar reports a relationship with Hookipa Biotech GmbH that includes consulting or advisory. The other authors declare that they have no known competing financial interests or personal relationships that could have appeared to influence the work reported in this paper.

The authors wish to acknowledge the CTOT-20 and CTOT-22 consortium sites with investigators and research coordinators for their instrumental effort toward patient enrollment, data collection, and sample collection and processing. Duke University: John M. Reynolds (principal investigator (PI)), Katelyn Arroyo, Erika Bush, Dongfeng Chen, Courtney Frankel, Annette Jackson, Fran Kelly, Allan Kirk, Stuart Knechtle, Justin Magin, Andrew Nagler, Megan Neely, Robyn Osborne, Scott Palmer, Elizabeth Pavlisko, Laurie Snyder, Jamie Todd, Daniel Turner, Jeremy Weber. University of Toronto: Lianne Singer (PI), Iva Avramov, Cecilia Chaparro, Noori Chowdhury, Marcelo Cuesta, Victor Ferreira, Atul Humar, Shahid Husain, David Hwang, Anam Islam, Stephen Juvet, Shaf Keshavjee, Deepali Kumar, Tereza Martinu, Max Niit, Dmitry Rozenberg, Alison Tian, Jussi Tikkanen, Kathryn Tinckam. Johns Hopkins University: Pali Shah (PI), Robin Avery, Maria Bettinotti, Peter Illei, Joby Mathew, Christian Merlo, Jonathan Orens, Jonathan Schenck. University of California, Los Angeles: John Belperio (PI), Eileen Callahan, Ariss DerHovanessian, Paul Lopez, Joseph Lynch III, Elman Punzalan, Elaine Reed, David Sayah, Michael Shino, Dean Wallace, Samuel Weigt. Cleveland Clinic: Marie Budev (PI), Valeria Arrosi, Adarsh Conjeevaram, Carol Farver, Stuart Houltham, Debra Kohn, Bette Maierson, Valerie Shaner, Wayne Tsuang, Aiwen Zhang. Duke Clinical Research Institute: Jerry Kirchner. Rho, Inc.: Brian Armstrong, Michele Cosgrove, David Ikle, Karen Kesler, Heather Kopetskie, Meghan McGinn, Michele Martin, Michelle Sever. NIAID: Julia Goldstein, Yvonne Morrison, Mark Robien, Nikki Williams.

We wish to acknowledge funding support for CTOT-20 and CTOT-22 (NIH National Institute of Allergy and Infectious Disease, U01AI113315), the Cystic Fibrosis Foundation (awarded to S.M.P., PALMER19AB0), and T32 Training Grant (awarded to M.J.H., 2T32AI052077-16A1).
