# Supplementary material for: Evaluating longitudinal cytomegalovirus-specific humoral immune responses and association with DNAemia risk in seropositive lung transplant recipients
Source: JHLT Open. 2024 May 28;5:100113. doi: 10.1016/j.jhlto.2024.100113 (PMC11935385; doi:10.1016/j.jhlto.2024.100113)
Supplement: Supplementary file 1 — Supplementary material [file mmc1.docx]

**Supplemental Figures**

**
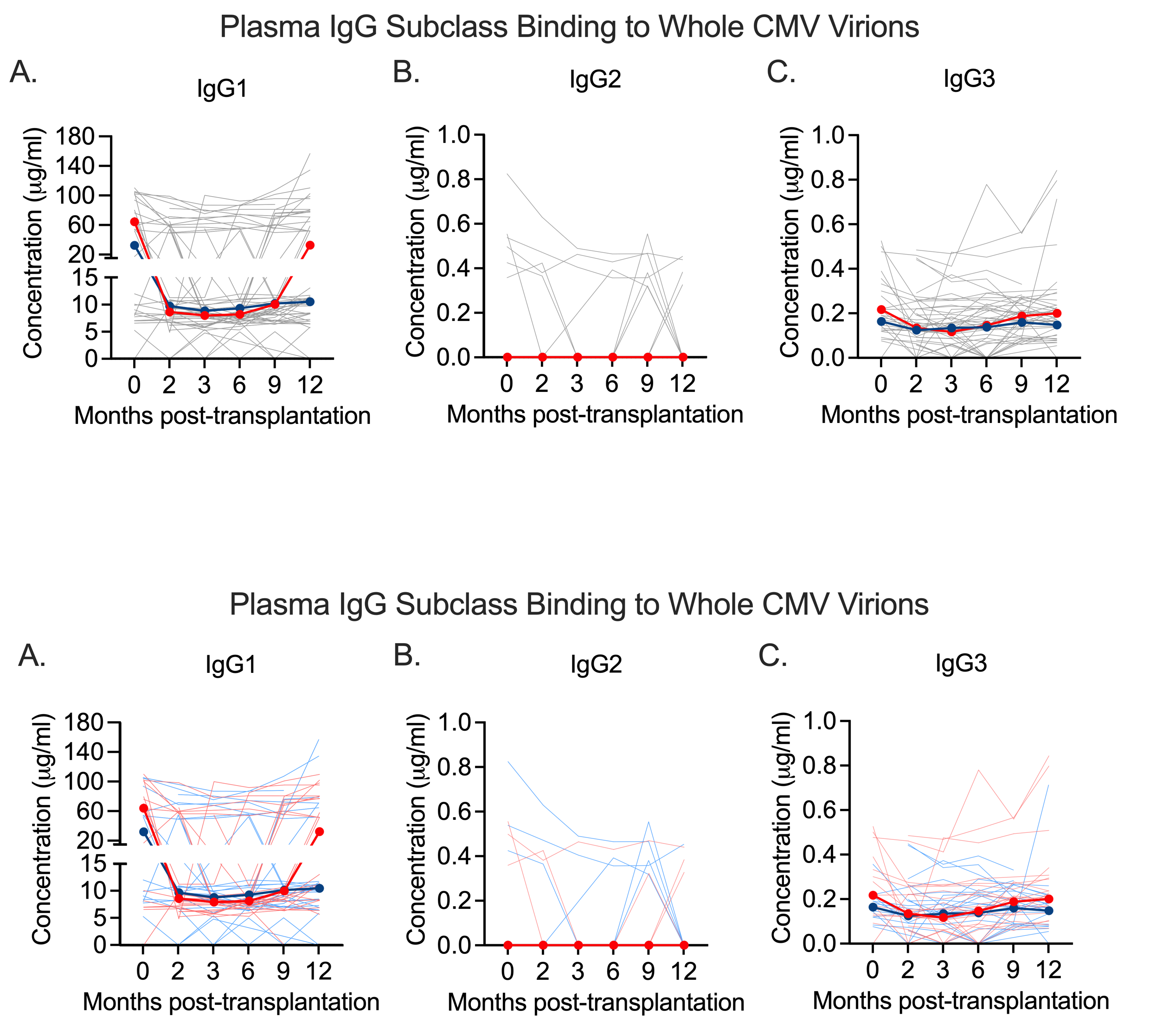
**

**Figure S1.** Whole AD169r virion-specific plasma (A) IgG1, (B) IgG2, and (C) IgG3 concentration in LT recipients who did not develop CMV DNAemia (blue) and who did develop CMV DNAemia (red) within 15 months of stopping antiviral prophylaxis. Thin lines show individual patient data, thick red and blue lines indicate median response. Concentration was calculated based on standard curves using known concentrations of IgG subclass protein isolated from human myeloma plasma. IgG4 was also measured but not detected.

**
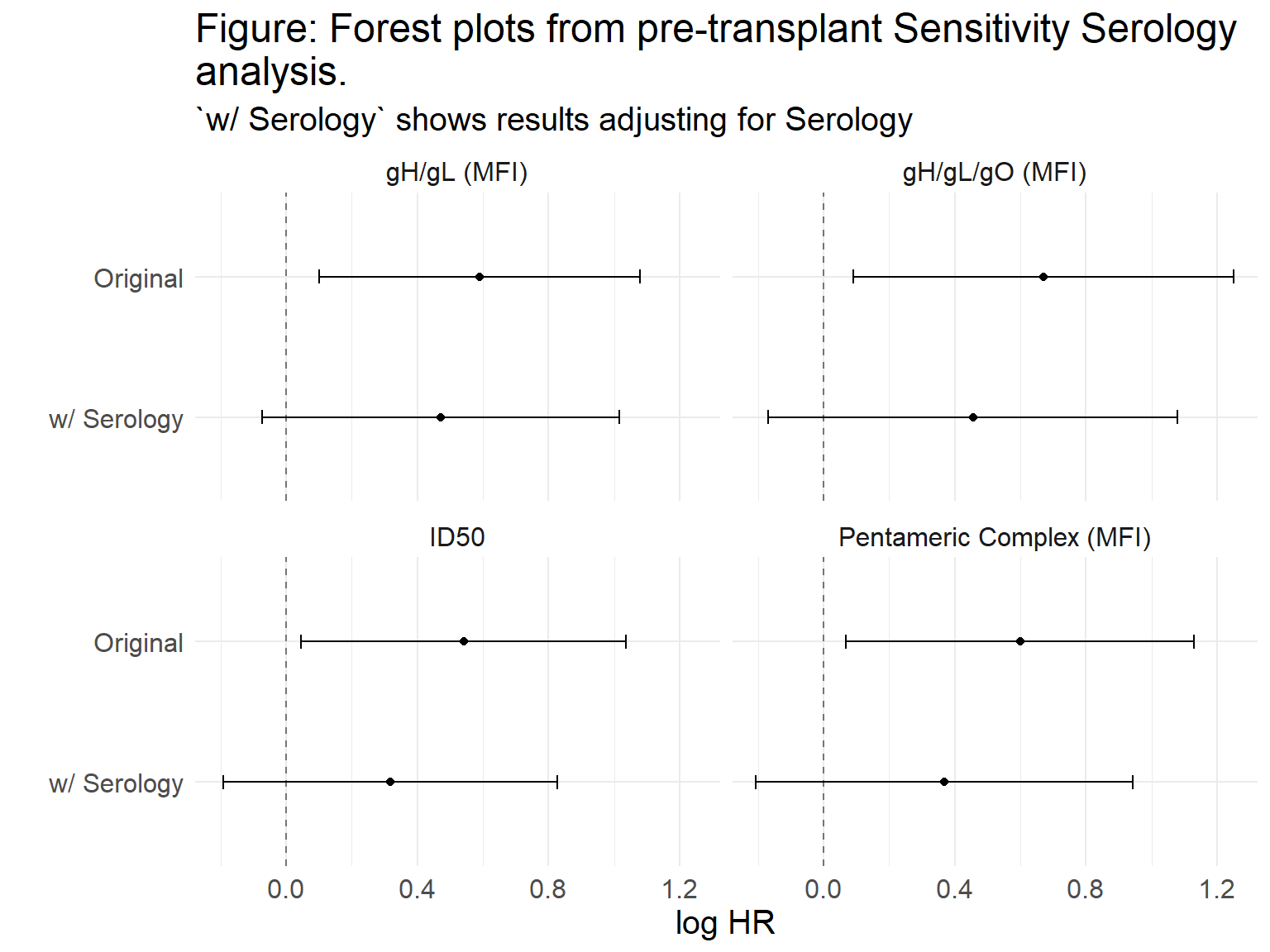
**

**Figure S2.** Forest plots from pre-transplant sensitivity analysis adjusting for donor CMV serostatus.

**Supplemental Tables**

**Table S1. Discovery cohort results from Cox-proportional hazards model.**

| **Variable** | **Log HR estimate** | **SE** | **P-value** | **FDR p-value** |
| --- | --- | --- | --- | --- |
| gH/gL (MFI) | 0.5900 | 0.250 | 0.0184 | 0.122 |
| gH/gL/gO (MFI) | 0.6700 | 0.296 | 0.0237 | 0.122 |
| Pentameric Complex (MFI) | 0.5990 | 0.271 | 0.0271 | 0.122 |
| Epithelial Neutralization (ID50) | 0.5410 | 0.253 | 0.0325 | 0.122 |
| IgG3 Concentration (µg/ml) | 0.3670 | 0.257 | 0.1530 | 0.374 |
| IgG binding to AD169r (AUC) | 0.3650 | 0.266 | 0.1690 | 0.374 |
| gB Ectodomain (MFI) | 0.3580 | 0.264 | 0.1740 | 0.374 |
| Fibroblast Neutralization (ID50) | 0.2290 | 0.220 | 0.2970 | 0.556 |
| Relative Avidity Index (%)* | -0.3290 | 0.354 | 0.3520 | 0.587 |
| Phagocytosis Score | -0.1850 | 0.235 | 0.4310 | 0.642 |
| ADCC (%) | 0.1820 | 0.253 | 0.4710 | 0.642 |
| IgG2 Concentration (µg/ml) | -0.1320 | 0.281 | 0.6380 | 0.798 |
| IgG1 Concentration (µg/ml) | 0.1030 | 0.269 | 0.7010 | 0.809 |
| IgM binding to AD169r (AUC) | -0.0593 | 0.279 | 0.8320 | 0.891 |
| gB-AD2 (AUC) | 0.0328 | 0.257 | 0.8980 | 0.898 |

**Table S2. Results from exploratory analysis of change from pre-transplant to early post-transplant responses.**

| **Variable** | **Log HR estimate** | **SE** | **P-value** | **FDR p-value** |
| --- | --- | --- | --- | --- |
| HFF Neutralization | -0.9420 | 0.433 | 0.0296 | 0.445 |
| ARPE Neutralization | -0.8940 | 0.501 | 0.0744 | 0.558 |
| IgG Whole Virion ELISA | -1.3400 | 0.904 | 0.1390 | 0.642 |
| IgG2 Concentration (µg/ml) | -4.7000 | 3.440 | 0.1710 | 0.642 |
| Pentameric Complex (MFI) | -0.5250 | 0.460 | 0.2530 | 0.760 |
| gB-AD2 (AUC) | -0.6630 | 0.707 | 0.3490 | 0.777 |
| gH/gL/gO (MFI) | -0.3710 | 0.425 | 0.3820 | 0.777 |
| ADCC (%) | -0.4040 | 0.495 | 0.4140 | 0.777 |
| Relative Avidity Index (%) | 0.8340 | 1.210 | 0.4910 | 0.818 |
| IgG1 Concentration (µg/ml) | 0.1010 | 0.238 | 0.6700 | 0.927 |
| gB ectodomain (MFI) | -0.1640 | 0.425 | 0.7000 | 0.927 |
| Phagocytosis Score | 0.4480 | 1.360 | 0.7410 | 0.927 |
| IgM binding to AD169r (AUC) | -0.1290 | 0.975 | 0.8950 | 0.965 |
| gH/gL (MFI) | 0.0137 | 0.177 | 0.9380 | 0.965 |
| IgG3 Concentration (µg/ml) | -0.1110 | 2.510 | 0.9650 | 0.965 |

**Table S3. Validation cohort results from Cox-proportional hazards model.**

| **Variable** | **Log HR estimate** | **SE** | **P-value** |
| --- | --- | --- | --- |
| PC | -0.1370 | 0.219 | 0.531 |
| gH/gL | -0.1340 | 0.225 | 0.551 |
| gH/gL/gO | -0.0721 | 0.218 | 0.740 |
| ARPE Neutralization | 0.0332 | 0.218 | 0.879 |
